# Total hip arthroplasty in patients with chronic liver disease: A systematic review

**DOI:** 10.1051/sicotj/2019037

**Published:** 2019-11-04

**Authors:** Elliot Onochie, Babar Kayani, Sebastian Dawson-Bowling, Steven Millington, Pramod Achan, Sammy Hanna

**Affiliations:** Department of Trauma and Orthopaedic Surgery, Royal London Hospital, Barts Health NHS Trust Whitechapel Road London E1 1BB UK

**Keywords:** Hip arthroplasty, Hip replacement, Liver disease, Cirrhosis, Outcomes, Complications

## Abstract

*Introduction*: Chronic liver disease (CLD) is a significant and increasingly prevalent co-morbidity in patients undergoing total hip arthroplasty (THA). These patients may develop metabolic bone disease (MBD) and systemic dysfunction, which pose challenges to THA surgery. This systematic review of literature aims to examine clinical outcomes and complications in patients with CLD undergoing THA and provide evidence-based approaches as to the optimization of their perioperative care.

*Methods*: A Pubmed search was performed, identifying eight studies on 28 514 THAs for inclusion. Two additional studies reported on 44 patients undergoing THA post liver transplant. These were reviewed separately.

*Results*: Increased early perioperative complications are reported recurrently. Review of long-term complications demonstrates an increased postoperative infection rate of 0.5% (*p* < 0.001) and perioperative mortality of 4.1% (*p* < 0.001). The need for revision surgery is more frequent at 4% (*p* < 0.001). Aetiology of need for revision surgery included; periprosthestic infection (70%), aseptic loosening (13%), instability (13%), periprosthetic fracture (2%) and liner wear (2%). THA in patients with liver transplants seems to offer functional improvement; however, no studies have formally assessed functional outcomes in the patient with active CLD.

*Discussion*: A multidisciplinary perioperative approach is suggested in order to minimize increased complication risks. Specific measures include optimizing haemoglobin and taking measures to reduce infection. This review also highlights gaps in available literature and guides future research to appraise functional outcomes, further detail long-term failure reasons and study any differences in outcomes and complications based on the range of operative approaches and available implant choices.

## Introduction

Chronic liver disease (CLD) is the fifth most common cause of mortality worldwide and its prevalence is increasing [1]. Hepatitis B and C viruses are the most common causes of CLD. Other causes include alcohol, drugs, hereditary and autoimmune diseases. Improvements in medical care have meant that patients with CLD are also surviving longer. Total hip arthroplasty (THA) is indicated to treat debilitating symptoms of hip osteoarthritis, including in those patients with CLD, this following lifestyle modifications and medical management. Several causes of CLD such as Sarcoidosis and Haemochromatosis are themselves associated with joint pathology, which may require THA. Patients whose CLD is secondary to chronic alcohol excess, or who are on long-term corticosteroids to treat CLD, are at an increased risk of developing avascular necrosis of the femoral head, which again may necessitate THA.

Performing THA in patients with CLD is challenging as the disease process induces biological and structural changes in bone, termed metabolic bone disease (MBD), whilst there is also systemic dysfunction [2]. The precise aetiology of bone disorders is thought to differ across the various causes of CLD, and indeed most pathological processes described are still somewhat conjectural [3]. MBD leads to osteopaenia and osteoporosis [4], whilst abnormal bone remodelling leads to bowing of the proximal femur with thinning of the cortices and widening of the medullary canal [5]. These morphological changes increase the technical challenges of performing THA. In press-fit implants, poor bone quality and unusual morphology means an increased risk of intraoperative fractures, subsidence of the femoral stem and postoperative periprosthestic fractures [6]. In cemented implants, these abnormalities may lead to inadequate cement mantles and an increased risk of aseptic loosening [7]. Coagulopathies in CLD may impair visualization of the surgical field, challenging surgical approach, implant positioning and wound closure. Coagulopathies increase intraoperative blood loss and also compromise the ability to achieve well-prepared, dry bone for bone-cement interdigitation. The use of tranexamic acid in THA has been shown to reduce bleeding without increasing thromboembolic risk [8]. Acetabular THA components have shown good results with both cemented and uncemented techniques in osteoporotic bone [9]. Finally, diminished hepatic biosynthetic and reticulo-endothelial capabilities together with depleted nutritional reserves, increase the risk of poor wound healing, superficial and deep infections.

With regard to the CLD patient, studies in English literature have reviewed surgical outcomes when performing general surgeries, and cumulating mixed arthoplasty cases, with reports of increased morbidity and mortality [10]. However, there are limited data in English literature on the specific risks for CLD patients undergoing THA. The objective of this study was to gather and systematically review available evidence in this area, summating the risks that a medical team must be aware of when considering THA in the patient with CLD. In addition, this review aims to evaluate functional outcomes in this patient group to assess whether the known technical challenges in this patient group are being successfully overcome.

## Materials and methods

### Search strategy

Databases PUBMED, MEDLINE and EMBASE were searched to identify relevant studies in English literature that addressed the results of THA in patients with CLD between 1980 and August 2019. This was performed in line with the PRISMA statement. Keywords used for the searches were “hip arthroplasty” OR “total hip arthroplasty” OR “total hip replacement” AND “Chronic Liver disease” OR “Liver Failure” OR “Cirrhosis” OR “Hepatitis”. The bibliographies of included studies and relevant foundation materials were reviewed judiciously to identify any supplementary studies for the review and for pertinent background materials

### Eligibility criteria

**Figure 1:**
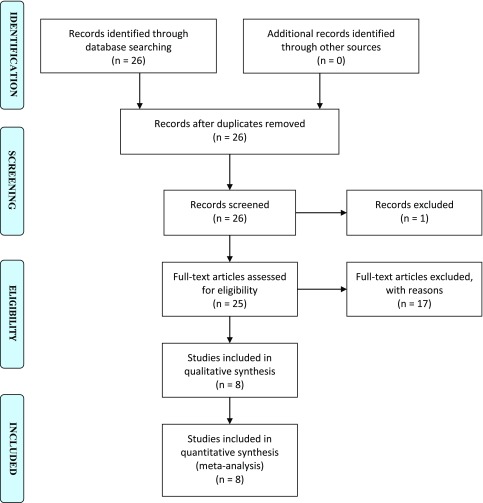
PRISMA flowchart illustrating the search strategy, number of records screened and included.

Inclusion criteria included all papers, describing the results of THA in patients with CLD published in the English language. Isolated case reports/series with five or less patients were excluded. The included articles met the PICO criteria (Population, Intervention, Comparison and Outcomes) for systematic reviews. Figure 1 is the PRISMA flowchart illustrating the systematic search and screening strategy resulting in the final number of records included.

### Data extraction

One reviewer extracted data through a standardized data collection form, and then another reviewer checked the data for accuracy. Any issues flagged up, or discrepancies in results were resolved by discussion. Data on the number of patients, age, follow-up period, type of implant, type of fixation, complications, re-operations, revision rate and functional outcomes were extracted and entered in a spreadsheet.

### Statistical analysis

All analyses compared CLD and non-CLD (control) patients, and all outcomes were binary in nature. The Chi-square test was used to compare groups for the majority of outcomes. The exception was for outcomes where the dataset was small in which Fisher’s exact test was performed. A *p* value of <0.05 was considered statistically significant.

## Results

### Search results

A total of 26 relevant article titles were identified. After application of eligibility criteria described, eight studies [11–18] qualified for inclusion. Two further studies [19, 20] reported on outcomes of THA in patients post liver transplantation for CLD, and this was deemed an interesting group for comment separately.

### Quality assessment

The included studies were small-to-large size retrospective case series (*n* = 19–27 401). The range of follow-up in the studies was 1–144 months. There was a significant heterogeneity between studies in terms of outcome recording.

### Cohort characteristics

The studies included 28 514 THAs performed in patients with a mean age of 57.3 years and mean follow up period of 13.5 months (range 1–144 months). Only two studies [12, 17] documented the types of implant used. There were an additional 44 THAs performed in patients post liver transplant with a mean age of 51.7 and mean follow up of 40.3 months [19, 20] 42 uncemented THAs (95%) and two cemented THAs (5%) were included in this subgroup of patients.

### Outcome analysis

#### Functional outcome

Both studies in patients with liver transplant patients reported significant improvements in patient satisfaction and hip function following THA [19, 20]. The remaining studies did not report on functional outcome scores following THA.

#### Aseptic loosening

Two studies reported on rates of aseptic loosening [12, 17]. There was an increased risk of aseptic loosening at 7% (6/85 THAs) compared with 0% in controls (*p* = 0.03). There was no comment on the mean timing of implant loosening. In patients with liver transplantation, there was one case of aseptic loosening in 44 THAs (2%), this at 39 months of follow-up. Further stratification based on cemented or uncemented prostheses was not recorded.

#### Revisions rate

Six studies reported on implant failure and revision rates [11–13, 16–18]. There was an increased rate of revision surgery at 4% (46/1083 THAs) compared with 0.2% in controls (*p* < 0.001). Time to implant failure was not reported. Reasons for implant failure included periprosthestic infection or septic loosening in 70% (*n* = 32), aseptic loosening in 13% (*n* = 6), instability in 13% (*n* = 6), periprosthetic fracture in 2% (*n* = 1), and polyethylene liner wear with osteolysis in 2% (*n* = 1). In liver transplant patients, implant failure occurred in three of the 44 patients (7%) at a mean time of 7.1 months. In these patients, implant failure secondary to instability with dislocation occurred in 67% (*n* = 2), and aseptic loosening in 33% (*n* = 1). Further stratification of implant failure based on the type of implant or revision surgery was not recorded.

#### Infection rate

Infection rate was reported in seven studies [12–18], which included 28 495 THAs. There was an increased infection rate in CLD patients, at 0.5% (range 0.3%–15.4%) compared with 0.15% in controls (*p* < 0.001). Two studies recorded separate infection rates for elective arthroplasty cases *versus* emergency cases [13, 18]. This amounted to 954 THAs, including 803 elective cases and 151 urgent procedures. There was an increased mean infection rate for elective cases at 4.1% (*n* = 33, *p* < 0.001) and for urgent cases at 8.6% (*n* = 13, *p* < 0.001). In patients with liver transplants, the mean infection rate was 1% [19, 20]

#### Mortality

Perioperative mortality rates were documented in five studies [11–14, 18] amounting to 1048 THAs. The mean perioperative mortality rate was increased in CLD patients at 4.1% compared with 0.2% in controls (*p* < 0.001). No perioperative mortality was reported in patients with liver transplants.

Table 1 summarizes the demographics and key findings of each study included in this systematic review.

**Table 1:** Demographics and key findings of studies included in this systematic review.

Study, Country	Age (mean)	Follow up (mean) months	No. of THAs	Revision rate (%)	Mortality rate (%)	Infection rate (%)
Cohen [11], USA	Elective: 65.7	1	19	5	5.3	–
	Urgent: 68.4					
Hsieh [12], Taiwan	55.2	84	45	37.8	11.1	24.4
Jiang [13], USA	62.3	6	878	1.2	1.9	4.0
Moon [14], Korea	60	1	30	–	6.7	10.0
Newman [15], USA	57.1	Until discharge	27 401	–	–	0.3
Orozco [16], USA	59	35	25	–	–	8.0
Pour [17], USA	55	101	40	8	–	15.0
Seol [18], South Korea	Elective: 61.9	Until discharge	76	Elective 13.5	3.9	14.5
	Urgent: 75.5					

## Discussion

Anecdotal evidence suggests that THA in patients with CLD is a generally successful procedure. However, this review highlights that there are no objective data within English literature that documents functional outcomes in this patient group, despite the fact that it is accepted that there are increased technical challenges. Indeed this study has found increased infection rates, mortality rates, rates of revision surgery and aseptic loosening in patients with CLD undergoing THA.

Long-term outcome measures are somewhat heterogeneously reported in current literature. These were examined during this study. This review found a revision surgery rate of 4%, with the majority of revisions being for periprosthetic infection/septic loosening of prosthesis (70%). The National Joint Registry (NJR) of England and Wales in 2016 reports a revision rate of 2.6% in THAs, and accordingly the suggestion is that THA revision rates are higher in patients with CLD. In addition, the most common underlying reason for revisions in NJR data was aseptic loosening at 24.2%, with infection only accounting for 13.8%. The limited numbers and heterogeneity of reporting, limit the ability to draw conclusions; however, there is a suggestion that infection may play a larger role in THA failures in patients with CLD. When considering THA in liver transplant patients, functional outcomes were shown to improve following surgery, with a 2% incidence of aseptic loosening, and a 7% need for revision surgery. Supporting this, Levitsky et al. (2003) [21] conducted a small review of arthroplasty cases in liver transplant patients (eight knee, three hip and one ankle). They found no deaths or major complications. These positive trends offer a faint suggestion that some aspects of MBD associated with CLD might be reversed after liver transplantation; however, the small numbers mean that drawing firm conclusions is not advisable. Indeed Cavanaugh et al. (2015) [22] conducted a retrospective study between 1993 and 2011 of 787 liver transplant patients who had undergone THA or Total Knee Arthroplasty (TKA) finding a higher risk of surgical site infection, renal and cardiorespiratory complications.

The inferences of this review are that there is an increased risk of general surgical and medical complications in patients with CLD undergoing THA. These findings are supported by previous research that has reviewed surgical outcomes in patients with CLD. Ziser et al. (1999) [10] conducted a review of 733 mixed surgeries at the Mayo clinic in 1999. This study reported significantly increased perioperative complications at 30.1%, increased mortality rate at 11.6%, and noted increased mortality with higher Child’s Pugh scores. The study also included a small number of hip and pelvic surgeries, and showed that these patients had significantly higher complication rates than other types of surgery (53% *vs*. 29.4%, *p* = 0.008). There are several studies that have looked at the outcomes of arthroplasty in patients with CLD, when combining THA and TKA. Deleuran et al. (2015) [23], retrospectively reviewed 363 THA and TKA cases in patients with CLD between 1995 and 2011. Patients with CLD had increased odds of mortality within 30 days (OR 3.9, 95% CI 1.5–10), deep infection (3.1% *vs*. 1.4%), and need for revision surgery (3.7% *vs*. 1.7%) compared to the control group. Tiberi et al. (2014) [24] reviewed clinical outcomes of 115 THA and TKA cases in patients with CLD between 2000 and 2012. This study showed patients with CLD had increased risk of urinary tract infection (*p* < 0.01), acute kidney injury (*p* < 0.03), need for transfusion (*p* < 0.01), dislocation (*p* = 0.01), infection (*p* = 0.02), 90-day revision surgery (*p* = 0.04) and 1 year mortality (*p* = 0.01) compared to the matched control group. Poultsides et al. (2013) [25] retrospectively reviewed 412 356 THA and 784 335 TKA between 1998 and 2007. Liver disease was found to be an independent risk factor for developing surgical site infection (OR = 2.53, *p* = 0.0001).

Worldwide, increasing numbers of THAs are being performed annually. It is reasonable to infer that arthroplasty surgeons will be performing THAs in patients with CLD with increasing frequency. It is thus essential to appreciate the medical and surgical issues unique to this patient-group, and particularly how to optimize controllable factors. Patel (1999) [26] discusses in detail systematic approaches to assess and optimize the patient with CLD for surgery. A multi-disciplinary approach is recommended. In line with this paper and other relevant evidence, the following pre-, intra- and post-operative considerations should be addressed:

### Pre-operative considerations/requirements

Advice may be sought from haematologists and/or hepatologists as to optimization strategies. The input of microbioloists may be sought on an individual case basis. Preoperative workup should aim to optimize haemoglobin levels. The cause for anaemia should be assessed and treated accordingly. Treatment may include nutritional supplementation such as with iron, or Erythropoietin where there is anaemia of chronic disease [27]. Pre-operative blood transfusion should not be considered a first-line option. It is important to obtain and review good quality radiographic studies of the pelvis and femur to assess bone morphology and quality, and plan surgery including selection of the most appropriate implants. Bisphosphonates have been shown to reduce periprosthetic bone loss and improve implant integration in those with osteoporotic bone [28] and thus should be considered.

### Intra-operative considerations

Surgery should be carried out with a focus on minimizing blood loss with vigilant haemostasis and the use of tranexamic acid [8]. Implant choice in THA in patients with CLD is complex. In osteoporotic bone, the surgeon may consider cemented femoral implants to reduce intraoperative fracture rates and aseptic loosening. With uncemented implants, the surgeon must appreciate general recommendations for implant preparation and fixation in osteoporotic bone.

This includes achieving good rim fit and using acetabular screws to enhance fixation in uncemented shells, and cautious femoral preparation and sizing choices.

### Postoperative considerations

Vigilant clinical assessment should be made with particular watchfulness for bleeding and infection as well other medical complications. Long-term follow up should include careful clinical and radiographic review for prosthesis loosening.

### Limitations

The results of this review must be interpreted with the limitations of this study in mind. All of the studies included in this review article are retrospective studies with their inherent limitations. There is non-uniform reporting of long-term complications, with some studies only reviewing short-term measures. Subgroup analysis has not been performed and confounding variables may have affected the outcomes recorded, for example, the use of immunosuppressant medication or steroids. With only two studies reporting on the type of implant used, it was not possible to draw conclusions or make recommendations as to the optimal implant properties for patients with CLD. The extent of CLD was also not stratified. Despite these limitations, this systematic review provides timely and important information for the medical community in clinical decision making and offering informed patient choices.

### Recommendations for research

There is a need for further large studies on patients with CLD undergoing THA. It is important to review functional outcomes so that patients can be fully informed when undertaking the decision to proceed with THA. With the availability of radiographic classifications such as by Dorr et al. (1993), it would be useful to study whether these influence outcomes, and whether this can be related to implant choices.

## Conclusion

A multidisciplinary perioperative approach is recommended in order to minimize increased complication risks, in particular infection, mortality, aseptic loosening and need for revision surgery. Infection may need heightened consideration when trying to avoid the serious complication of need for revision surgery. This review guides future research to appraise functional outcomes of THA in patients with active CLD, further detail long-term failure reasons, and review any differences in outcomes and complications based on various operative approaches and available implants.

## Conflicts of interest

All the authors declare that they have no competing interests.

## References

[1] Scaglione S, Kliethermes S, Cao G, et al. (2015) The epidemiology of cirrhosis in the United States: A population-based study. J Clin Gastroenterol 49(8), 690–696. 25291348 10.1097/MCG.0000000000000208

[2] Goral V, Simsek M, Mete N (2010) Hepatic osteodystrophy and liver cirrhosis. World J Gastroenterol 16(13), 1639–1643. 20355242 10.3748/wjg.v16.i13.1639 PMC2848372

[3] Handzlik-Orlik G, Holecki M, Wilczyński K, et al. (2016) Osteoporosis in liver disease: Pathogenesis and management. Ther Adv Endocrinol Metab 7(3), 128–135. 27293541 10.1177/2042018816641351 PMC4892399

[4] Luxon B (2011) Bone disorders in chronic liver diseases. Curr Gastroenterol Rep 13, 40–48. 21104208 10.1007/s11894-010-0166-4

[5] Dorr LD, Faugere MC, Mackel AM, et al. (1993) Structural and cellular assessment of bone quality of proximal femur. Bone 14(3), 231–242. 8363862 10.1016/8756-3282(93)90146-2

[6] Thien TM, Chatziagorou G, Garellick G, et al. (2014) Periprosthetic femoral fracture within two years after total hip replacement: Analysis of 437,629 operations in the nordic arthroplasty register association database. J Bone Jt Surg Am 96(19), e167. 10.2106/JBJS.M.00643 25274795

[7] Carli AV, Negus JJ, Haddad FS (2017) Periprosthetic femoral fractures and trying to avoid them: What is the contribution of femoral component design to the increased risk of periprosthetic femoral fracture? Bone Joint J 99-B(1 Supple A), 50–59. 28042119 10.1302/0301-620X.99B1.BJJ-2016-0220.R1

[8] Sukeik M, Alshryda S, Haddad FS, et al. (2011) Systematic review and meta-analysis of the use of tranexamic acid in total hip replacement. J Bone Joint Surg Br 93(1), 39–46. 21196541 10.1302/0301-620X.93B1.24984

[9] Hailer NP, Garellick G, Kärrholm J (2010) Uncemented and cemented primary total hip arthroplasty in the Swedish Hip Arthroplasty Register. Acta Orthop 81(1), 34–41. 20180715 10.3109/17453671003685400 PMC2856202

[10] Ziser A, Plevak DJ, Wiesner RH, et al. (1999) Morbidity and mortality in cirrhotic patients undergoing anesthesia and surgery. Anesthesiology 90(1), 42–53. 9915311 10.1097/00000542-199901000-00008

[11] Cohen SM, Te HS, Levitsky J (2005) Operative risk of total hip and knee arthroplasty in cirrhotic patients. J Arthroplast 20(4), 460–466. 10.1016/j.arth.2004.05.004 16124961

[12] Hsieh PH, Chen LH, Lee MS, et al. (2003) Hip arthroplasty in patients with cirrhosis of the liver. J Bone Joint Surg Br 85(6), 818–821. 12931797

[13] Jiang SL, Schairer WW, Bozic KJ (2014) Increased rates of periprosthetic joint infection in patients with cirrhosis undergoing total joint arthroplasty. Clin Orthop Relat Res 472(8), 2483–2491. 24711129 10.1007/s11999-014-3593-y PMC4079852

[14] Moon Y-W, Kim Y-S, Kwon S-Y, Kim S-Y, Lim S-J, Park Y-S (2007) Perioperative risk of hip arthroplasty in patients with cirrhotic liver disease. J Korean Med Sci 22(2), 223–226. 17449928 10.3346/jkms.2007.22.2.223 PMC2693586

[15] Newman JM, Schiltz NK, Mudd CD, et al. (2016) Impact of cirrhosis on resource use and inpatient complications in patients undergoing total knee and hip arthroplasty. J Arthroplast 31(11), 2395–2401. 10.1016/j.arth.2016.04.011 PMC5069119 27236746

[16] Orozco F, Post Z, Baxi O, et al. (2014) Fibrosis in hepatitis C patients predicts complications after elective total joint arthroplasty. J Arthroplast 29, 7–10. 10.1016/j.arth.2013.03.023 23648106

[17] Pour A, Matar W, Jafari M, et al. (2011) Total joint arthroplasty in patients with hepatitis C. J Bone Joint Surg Am 15, 1448–1454. 10.2106/JBJS.J.00219 21915551

[18] Seol YJ, Yoon TR, Lee DH, et al. (2017) Outcome analysis of hip or knee arthroplasty in patients with cirrhotic liver disease. J Orthop 14(1), 171–175. 28070149 10.1016/j.jor.2016.12.011 PMC5219598

[19] Aminata I, Lee SH, Chang JS, et al. (2012) Perioperative morbidity and mortality of total hip replacement in liver transplant recipients: A 7-year single-center experience. Transplantation 94(11), 1154–1159. 23089978 10.1097/TP.0b013e31826ec713

[20] Ledford CK, Watters TS, Wellman SS, et al. (2014) Risk versus reward: Total joint arthroplasty outcomes after various solid organ transplantations. J Arthroplast 29(8), 1548–1552. 10.1016/j.arth.2014.03.027 24768542

[21] Levitsky J, Te HS, Cohen SM (2003) The safety and outcome of joint replacement surgery in liver transplant recipients. Liver Transpl 9(4), 373–376. 12682889 10.1053/jlts.2003.50067

[22] Cavanaugh PK, Chen AF, Rasouli MR, et al. (2015) Total joint arthroplasty in transplant recipients: In-hospital adverse outcomes. J Arthroplast 30(5), 840–845. 10.1016/j.arth.2014.11.037 25540994

[23] Deleuran T, Vilstrup H, Overgaard S, et al. (2015) Cirrhosis patients have increased risk of complications after hip or knee arthroplasty. Acta Orthop 86(1), 108–113. 25238440 10.3109/17453674.2014.961397 PMC4366671

[24] Tiberi JV, Hansen V, El-Abbadi N, et al. (2014) Increased complication rates after hip and knee arthroplasty in patients with cirrhosis of the liver. Clin Orthop Relat Res 472(9), 2774–2778. 24993141 10.1007/s11999-014-3681-z PMC4117879

[25] Poultsides LA, Ma Y, Della Valle AG, et al. (2013) In-hospital surgical site infections after primary hip and knee arthroplasty – Incidence and risk factors. J Arthroplast 28(3), 385–389. 10.1016/j.arth.2012.06.027 23142444

[26] Patel T (1999) Surgery in the patient with liver disease. Mayo Clin Proc 74(6), 593–599. 10377935 10.4065/74.6.593

[27] Goodnough LT, Maniatis A, Earnshaw P, et al. (2011) Detection, evaluation and management of preoperative anaemia in the elective orthopaedic surgical patient: NATA guidelines. Br J Anaesth 106(1), 13–22. 21148637 10.1093/bja/aeq361 PMC3000629

[28] Sköldenberg OG, Salemyr MO, Bodén HS, et al. (2011) The effect of weekly risedronate on periprosthetic bone resorption following total hip arthroplasty: A randomized, double-blind, placebo-controlled trial. J Bone Jt Surg Am 93(20), 1857–1864. 10.2106/JBJS.J.01646 22012522

